# Magnesium Modified β-Tricalcium Phosphate Induces Cell Osteogenic Differentiation In Vitro and Bone Regeneration In Vivo

**DOI:** 10.3390/ijms23031717

**Published:** 2022-02-02

**Authors:** Eisner Salamanca, Yu-Hwa Pan, Ying-Sui Sun, Hao-Wen Hsueh, Odontuya Dorj, Wan-Ling Yao, Jerry Chin-Yi Lin, Nai-Chia Teng, Ikki Watanabe, Shinichi Abe, Yi-Fan Wu, Wei-Jen Chang

**Affiliations:** 1 School of Dentistry, College of Oral Medicine, Taipei Medical University, Taipei 11031, Taiwan; eisnergab@hotmail.com (E.S.); shalom.dc@msa.hinet.net (Y.-H.P.); lamenisocu@gmail.com (H.-W.H.); dorj.odontuya@gmail.com (O.D.); yaoyao06167@gmail.com (W.-L.Y.); drjerrylin@gmail.com (J.C.-Y.L.); dianaten@tmu.edu.tw (N.-C.T.); 2 Department of General Dentistry, Chang Gung Memorial Hospital, Taipei 10507, Taiwan; 3 Graduate Institute of Dental & Craniofacial Science, Chang Gung University, Taoyuan 33305, Taiwan; 4 School of Dentistry, College of Medicine, China Medical University, Taichung 40402, Taiwan; 5 School of Dental Technology, College of Oral Medicine, Taipei Medical University, Taipei 11031, Taiwan; yingsuisun@tmu.edu.tw; 6 Department of Dental Technology and Hygiene, Mongolian National University of Medical Sciences, Ulaanbaatar 14210, Mongolia; 7 Department of Oral Medicine, Infection and Immunity, Harvard School of Dental, Medicine, Boston, MA 02115, USA; 8 Dental Department, Taipei Medical University Hospital, Taipei 11031, Taiwan; 9 Department of Gerontology, Tokyo Medical and Dental University, Tokyo 113-8510, Japan; ikki.ore@tmd.ac.jp; 10 Department of Anatomy, Tokyo Dental College, Tokyo 101-0061, Japan; abesh@tdc.ac.jp; 11 Dental Department, Shuang-ho Hospital, Taipei Medical University, New Taipei City 23561, Taiwan

**Keywords:** β-tricalcium phosphate, magnesium ions, material characterization, osteogenic differentiation, bone regeneration, hydrothermal synthesis, dental research

## Abstract

In vitro, in vivo, and clinical studies have shown how the physicochemical and biological properties of β-tricalcium phosphate (β-TCP) work in bone regeneration. This study aimed to improve the properties of β-TCP by achieving optimum surface and bulk β-TCP chemical/physical properties through the hydrothermal addition of magnesium (Mg) and to later establish the biocompatibility of β-TCP/Mg for bone grafting and tissue engineering treatments. Multiple in vitro and in vivo analyses were used to complete β-TCP/Mg physicochemical and biological characterization. The addition of MgO brought about a modest rise in the number of β-TCP surface particles, indicating improvements in alkaline phosphatase (ALP) activity on day 21 (*p* < 0.05) and in the WST-1assay on all days (*p* < 0.05), with a corresponding increase in the upregulation of ALP and bone sialoprotein. SEM analyses stated that the surfaces of the β-TCP particles were not altered after the addition of Mg. Micro-CT and histomorphometric analysis from rabbit calvaria critical defects resulted in β-TCP/Mg managing to reform more new bone than the control defects and β-TCP control at 2, 6, and 8 weeks (* *p* ≤ 0.05, ** *p* ≤ 0.01, *** *p* ≤ 0.001, and **** *p* ≤ 0.0001). The hydrothermal addition of MgO to the β-TCP surfaces ameliorated its biocompatibility without altering its surface roughness resulting from the elemental composition while enhancing cell viability and proliferation, inducing more bone regeneration by osteoconduction in vivo and osteoblastic differentiation in vitro.

## 1. Introduction

In the dental field, the treatment of pathological conditions such as periodontitis, traumatisms, and oral cancer can lead to different types of bone defects. For example, the results of a radiographic evaluation showed that the prevalence and distribution of bone defects were linked to chronic periodontitis. In addition, the authors found that the prevalence of bone defects was over 80% and that bone loss was higher in males than in females [1]. Oral cancer is the sixth most common malignancy worldwide [2].However, root fractures are responsible for only 0.5%–0.7% of all dentoalveolar cases in permanent dentition and are frequently accompanied by fractures of the alveolar bone. The restoration of these anterior dental traumas in young adults can be complex [3]. Even though oral cancer is not prevalent compared to other tumor entities, the condition generates significant mortality and morbidity in patients, specifically when discovered in the delayed stages of the disease [4]. When oral cancer is successfully treated, the treatment of bone defects becomes necessary. All of these bone defects can be treated with autografts, which are still considered the gold standard and are most used type of bone graft [5]. However, autografts have several drawbacks, including a limited supply and additional surgery to obtain the graft, with possible donor-site morbidity [5]. The above-mentioned obstacles can be avoided by using other types of bone grafts, such as biocompatible and bioresorbable alloplastic pure-phase β-TCP. With β-TCP, particle sizes that vary between 500 and 1000 μm with 65% porosity can improve osteoblastogenesis by ameliorating the capillary effect of the blood, which stimulates new bone regeneration by osteoconduction over a period of between 4 and 12 months [6]. Nowadays, β-TCP is used in a range of bone surgeries in dentistry and medical orthopedics [7,8,9].

In recent years, the particular concentration of magnesium (Mg) ions has been found to play a pivotal role in new bone formation and regeneration. The addition of Mg ions is a simple yet effective method to enhance the osteogenesis and angiogenesis of bio-ceramic scaffolds and to ameliorate the evolution of biomaterials and bone tissue engineering scaffolds [10]. However, Mg can trigger an acute and unfavorable inflammatory response that can lead to the destruction of adjacent normal bone and the formation of fibrous tissue. [11]. In a more recent review article, the addition of Mg^2+^ to BCP was reported to lead to desirable physicochemical properties that could be observed in both in vitro and in vivo results, suggesting that Mg-doped biphasic calcium phosphate (Mg-BCP) is biocompatible and efficient as a bone substitute. Despite these promising outcomes, similar to other biomaterials for bone regeneration, the optimal physicochemical properties must be established. Further investigations are required regarding Mg-BCP applications in bone tissue engineering [12].

In daily dental practice, to enhance bone tissue engineering approaches for the various types of bone defects and to achieve the best surface and bulk material properties, the β-TCP and MgO synthesis method should be chosen carefully [13]. Having the ability to create crystalline phases that are not stable at the melting point, hydrothermal synthesis, also known as the hydrothermal method, can be used to synthesize crystals that depend on the solubility of minerals in hot water under high pressure [14]. This method has proven to be a suitable Mg-doping method at the phase transformation temperature of β-TCP ceramics [15]. However, minimal modifications in the sintering process of β-TCP can lead to different properties that ultimately can undermine results, such as new bone regeneration, when used for bone defect treatments [7]. 

One method of avoiding these different outcomes can be achieved by taking advantage of Mg, which can be adsorbed to a particle’s surface because of the colloidal interactions between the particles and present ions [16] that could be deposited in already-produced β-TCP particles used daily in clinical dental practice to improve its new bone formation capabilities without modification.

This study aimed to improve β-TCP properties by achieving optimum surface and bulk β-TCP chemical/physical properties through the hydrothermal addition of Mg and to later establish β-TCP/Mg biocompatibility for bone graft tissue engineering treatments.

## 2. Results 

### 2.1. Physicochemical Properties of β-TCP/Mg

**Figure 1:**
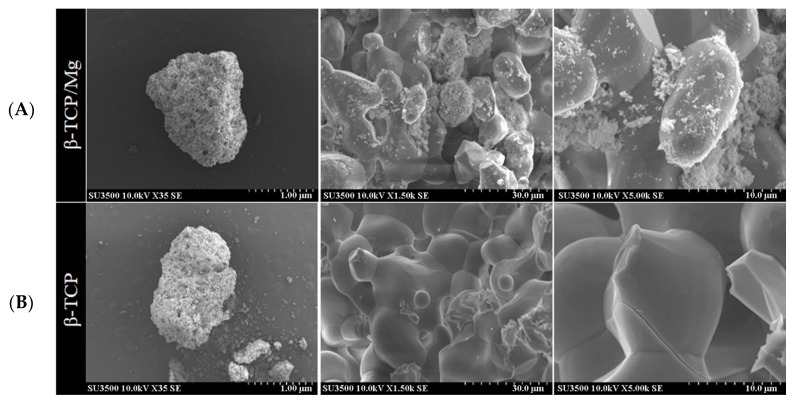
Scanning electron microscope images of the surface view of the obtained hydrothermally treated vs. nontreated β-TCP. (**A**) β-TCP/Mg apparent fusion of MgO ions with the β-TCP surface is observed. (**B**) β-TCP control.

The SEM results show that the β-TCP/Mg particles exhibited Mg on their surfaces while maintaining the similar roughness and morphology of the β-TCP control particle structures (Figure 1). 

In vitro, the EDS results demonstrated that a 1.4 weight percentage of Mg was uniformly distributed on the β-TCP/Mg surface while maintaining a 19.7 wt% P and slightly inferior 35.9 wt% Ca compared to the 20.8 wt% P and 39.2 wt% Ca found in the control β-TCP (Table 1). 

**Figure 2:**
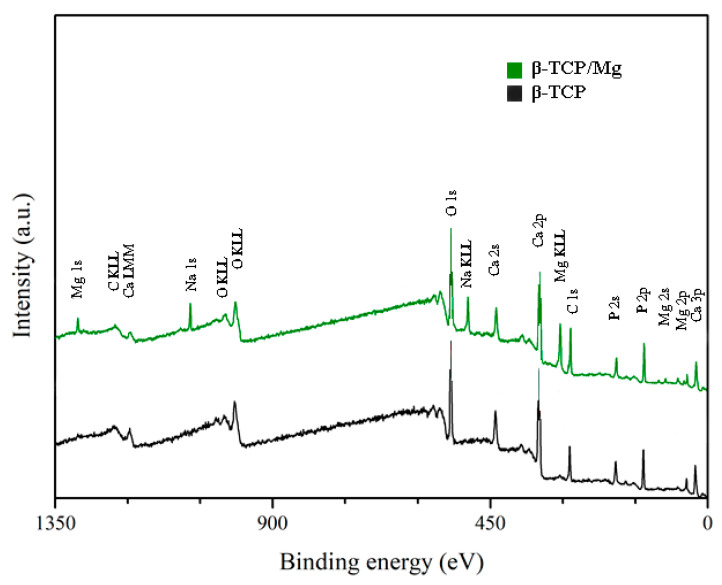
XPS spectra. Untreated beta-TCP and beta-TCP/Mg fragments reveal Ca, P, and O. Limited quantities of Mg were present in the β-TCP/Mg samples.

The XPS results exhibited no statistically significant differences between the Ca/P ratio of β-TCP/Mg particles (1.40) and that of β-TCP particles (1.38). However, both β-TCP particles had slightly lower Ca/P ratios than the previously described 1.5 stoichiometric TCP ratio [17]. The (O1s) mean value for β-TCP/Mg was 48.43%, and that for the β-TCP control was 49.74%. The values for the β-TCP control and β-TCP/Mg samples were 11.80% and 8.79% (P2p), respectively. At the same time, the Ca2p values were 12.36% in β-TCP/Mg and 16.36% in the β-TCP control (Table 2; Figure 2).

**Figure 3:**
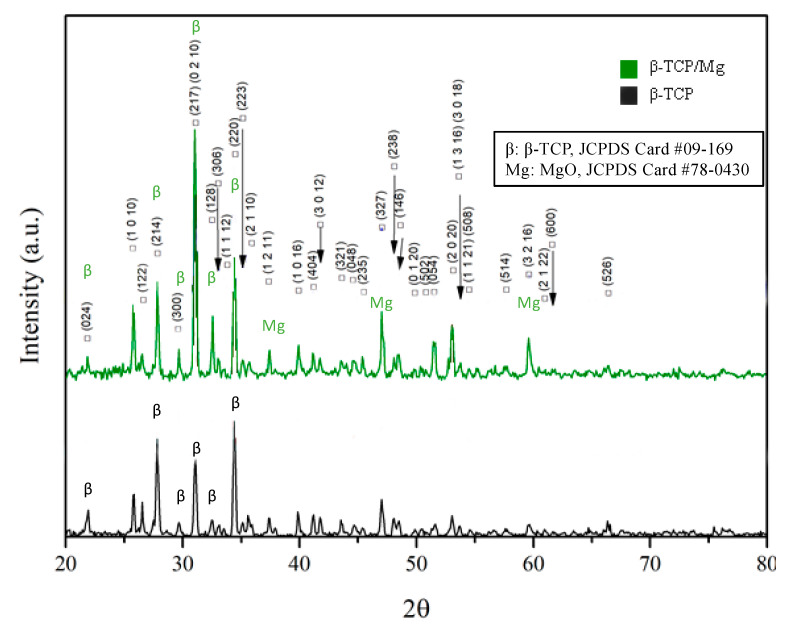
X-ray diffraction patterns of β-TCP/Mg and β-TCP control particles generating the same intense sharp-peak samples.

Figure 3 Illustrates that MgO incorporation had no significant effect on the lattice parameters detected by XRD in the β-TCP structure, contrary to what was found in a study where Mg^2+^ was doped at the phase transformation temperature of β-TCP ceramics. Nevertheless, the β-TCP/Mg showed diffraction patterns corresponding to the β-TCP (JCPDS file no. 09-169, International Center for Diffraction Data) and MgO reference peaks (JCPDS file no. 78-0430, International Center for Diffraction Data), indicating that the MgO powders were well-incorporated on β-TCP graft’s surface overall.

**Figure 4:**
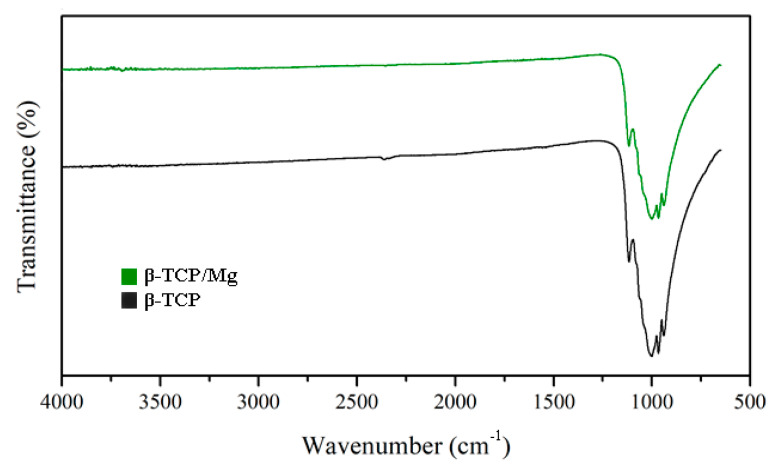
Beta-TCP/Mg and beta-TCP control have identical FTIR spectra absorption peak heights.

The FTIR results reinforced the XRD findings by presenting similar high-intensity peaks at the 1043 and 1120 cm^−1^ absorption bands in the β-TCP/Mg and untreated β-TCP particles (Figure 4). 

### 2.2. In Vitro Biocompatibility

**Figure 5:**
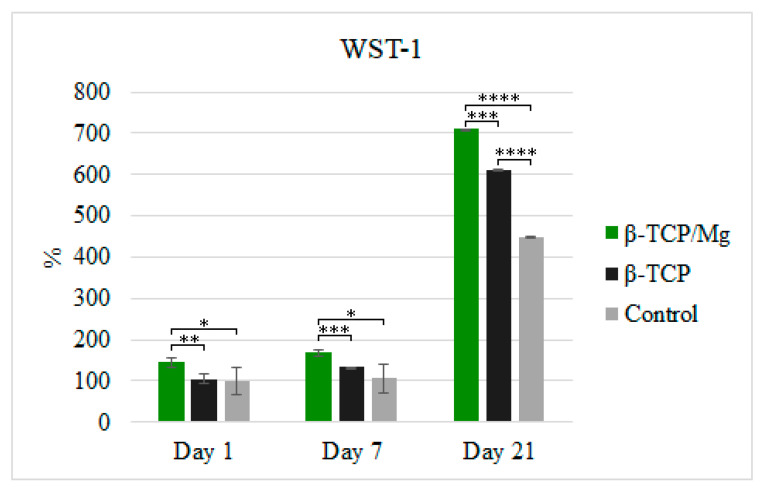
MG-63 cell proliferation measured using WST-1 on days 1, 7, and 21. * *p* ≤ 0.05, ** *p* ≤ 0.01, *** *p* ≤ 0.001, and **** *p* ≤ 0.0001.

The in vitro cytotoxicity and proliferation of MG-63 at 1, 7, and 21 days in the β-TCP/Mg and untreated β-TCP media behaved according to ISO10993-5. The results are shown in Figure 5. The percentages of cells cultivated in the media with the β-TCP/Mg particles were 144.45%, 168.92%, and 709.88% on days 1, 7, and 21, respectively. Conversely, MG-63 proliferation on the nontreated β-TCP was 105.22%, 132.84%, and 611.90% on days 1, 7, and 21, respectively. 

**Figure 6:**
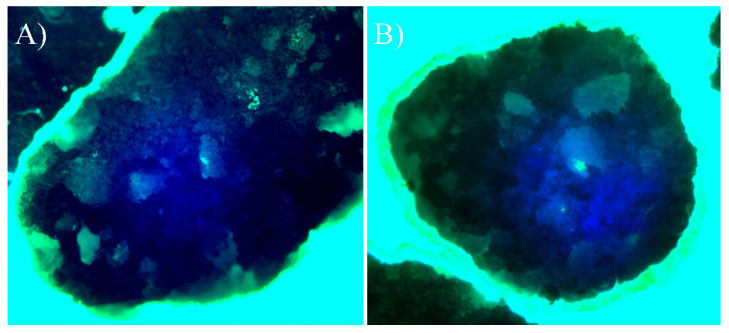
Direct cell culture with DAPI (4′,6-diamidino-2-phenylindole)/phalloidin fluorescent imaging at 20× after 24 h. (**A**) Beta-tricalcium phosphate; (**B**) Beta-tricalcium phosphate/Mg.

β-TCP/Mg improved MG-63 cell proliferation and displayed excellent biocompatibility with a statistically significant difference compared to the cells cultured with nontreated β-TCP and control cells (Figure 5). However, the cells cultured with untreated β-TCP had proliferated significantly more than control cells (*p* < 0.0001) by day 21. Additionally, when phalloidin/DAPI immunofluorescence staining was used, it was determined that the cells on β-TCP control and β-TCP/Mg proliferated and presented with a spindle shape and filopodial extensions (Figure 6).

**Figure 7:**
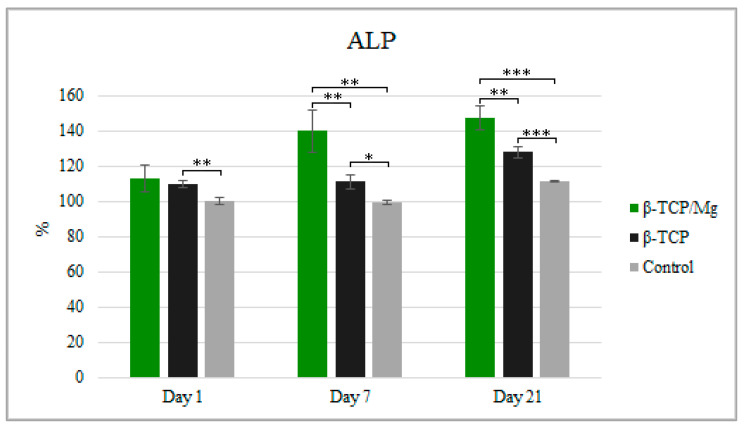
ALP activity of MG-63 after being cultured for 1, 7, and 21 days with the β-TCP/Mg, untreated β-TCP, or just DMEM as control. * *p* ≤ 0.05, ** *p* ≤ 0.01, and *** *p* ≤ 0.001.

Despite having significantly more cell proliferation, as observed in WST-1, the ALP test showed MG-63 cultivation with β-TCP/Mg, with 112.92% cultivation at 1 day, 139.95% cultivation at day 7, and 147.37% cultivation at day 21 when compared to the number of cells in the β-TCP/medium or in the medium alone after 21 days of culture. Furthermore, the cells produced higher ALP when cultured in the β-TCP/Mg medium than when cultured in the β-TCP control or in medium alone at 21 days (*p* < 0.05; Figure 7).

**Figure 8:**
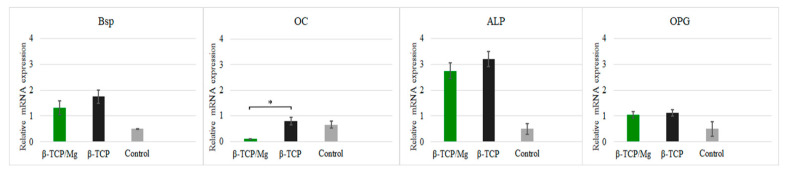
Relative expression BSP, OC, ALP, and OPG of MG-63 cells maintained in beta-tricalcium phosphate/Mg, beta-tricalcium phosphate, or just DMEM (control). * *p* ≤ 0.05.

Nonetheless, high concentrations of Mg ions reduced osteoblast differentiation and inhibited the expression of specific osteogenic markers [18], as was seen with the gene expression in this study (Figure 8). 

In vivo, the β-TCP/Mg particle grafts started creating new bone while degrading critical calvaria size defect healing in the New Zealand white rabbits, as seen in micro-CT results, from week 2 to week 7.

**Figure 9:**
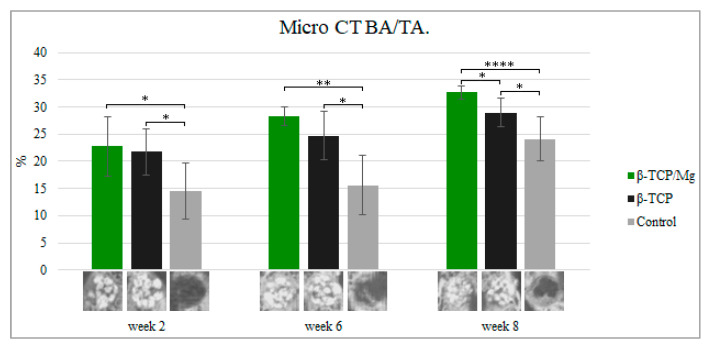
Micro-CT new bone area/tissue area formation, 40×. * *p* ≤ 0.05, ** *p* ≤ 0.01, and **** *p* ≤ 0.0001.

During week 2, new bone formation defects in the calvarial region packed with untreated β-TCP, β-TCP/Mg, and control regenerated 21.74% ± 4.24%, 22.73% ± 5.39%, and 14.54% ± 5.23%, respectively. The defects that were treated with beta-TCP control and beta-TCP/Mg were significantly superior in bone formation than in control defects (*p* < 0.05, Table 3, Figure 9).

Up to 28.36% ± 1.70% new bone regeneration was achieved by β-TCP/Mg during week 6, which was significantly higher than that observed for the β-TCP control (24.73% ± 4.41%) and control groups (15.58% ± 5.45%). Statistically significant differences were exhibited between both the beta-TCP/Mg and beta-TCP (*p* < 0.01) with β-TCP/Mg and the control group *(p* < 0.05) (Table 3, Figure 9).

At eight weeks, β-TCP/Mg induced 32.65% ± 1.24% bone regeneration. In addition, new bone formation was significantly more extensive in the β-TCP/Mg group than it was in the β-TCP control (28.98% ± 2.65%) or in the control group (24.1% ± 4.05%) (*p* < 0.05, Table 3, Figure 9).

Additionally, control defects did not close and maintained the space that they took up. In contrast, the β-TCP/Mg and untreated β-TCP particles managed to yield greater defect closure and space maintenance at eight weeks (Table 3, Figure 9).

### 2.3. In Vivo Biocompatibility

**Figure 10:**
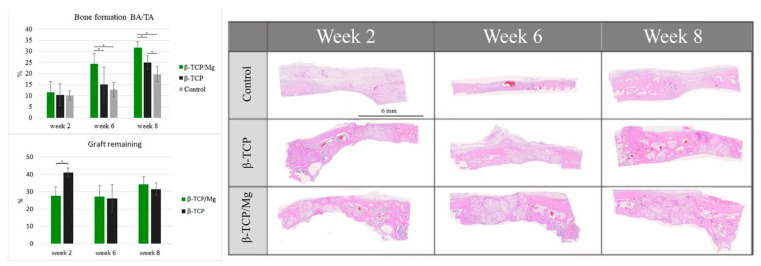
Histologic BA/TA new bone formation and remaining graft 40×. * *p* ≤ 0.05.

The control group regenerated 10.14% ± 2.11% new bone, which did not differ significantly from the 10.42% ± 4.89% of the β-TCP control group and the 11.48% ± 4.88% of the β-TCP/Mg group (*p* < 0.05) (Table 4, Figure 10). β-TCP/Mg had faster graft degradation, with 27.73 ± 12.50% graft remaining, while the β-TCP control had a lower graft resorption rate with 41.10 ± 6.80% (*p* < 0.05) (Figure 10).

On week 6, more additional new bone was found within the particles. The β-TCP control generated 15.07 ± 7.91% more new bone than the control group did: 12.73% ± 3.26%. Additionally, β-TCP/Mg regenerated 24.42% ± 4.53% new bone, which was greater than that of the control (*p* < 0.001) and β-TCP control (*p* < 0.05) defects (Table 4, Figure 10). Both β-TCP/Mg and β-TCP had similar graft resorption rates with 27.30 ± 14.84% and 26.20 ± 20.1%, respectively.

On week 8, the graft particles started reabsorbing while regenerating new bone formation, and bone maturation was able to be seen through defects because of the particle scaffold characteristics. The β-TCP/Mg (31.62% ± 3.03%) had better bone regeneration over the β-TCP control (*p* < 0.05) and the control (*p* < 0.001). Moreover, the β-TCP control group (24.94% ± 3.00%) had significantly higher amounts of new bone tissue than the control group (19.73% ± 3.37%, *p* < 0.05), as shown in Table 4 as well as in Figure 10. The graft resorption rate had a similar tendency to the one observed at week 6, with both grafts having no statistically significant differences, with β-TCP/Mg and β-TCP show rates of 34.25 ± 3.40% and 31.57 ± 3.94%, respectively.

For the critical defects that were treated with β-TCP/Mg (histologic new bone formation: 31.62% ± 3.03%; micro-CT new bone formation: 32.65 ± 1.24), the new bone area was 4% greater on average than those treated with the β-TCP control (histologic new bone formation: 24.94% ± 3.00%; micro-CT new bone formation: 28.98 ± 2.65) (*p* < 0.05, Table 3 and Table 4). 

## 3. Discussion

Calcium phosphate is used for tissue engineering due to its excellent bioactivity and reasonable biocompatibility with living tissues [19]. Hence, it is considered to be a remedy for fractured bones [20]. In the current study, we wanted to enhance the properties of β-TCP by achieving optimum surface and bulk β-TCP chemical/physical properties through the hydrothermal addition of Mg and to later establish β-TCP/Mg biocompatibility for bone graft tissue engineering. Furthermore, control β-TCP is already a commercial bone graft biomaterial that is used in dentistry for guided bone regeneration [21,22,23]. We agree that this commercial β-TCP, when compared to autogenous bone grafts, can produce similar improvements in sinus lifting and in two- to three-wall intrabony defects, among other bone defect regeneration treatments. Therefore, the use of β-TCP can be suggested when autogenous bone graft is in limited supply or not a possible option. Despite the good results of previous clinical studies, β-TCP can still be improved. One way to achieve this is by substituting Mg in calcium phosphate because of its potential role in the qualitative changes in the bone matrix, its indirect influence on mineral metabolism, its promotion of catalytic reactions, and its control of biological functions [24].

The surfaces of the β-TCP/Mg particles resembled those of the β-TCP control particles, showing typical phosphate ceramic surface roughness, which is used to stimulate cell attachment [25,26]. These findings were consistent with previous studies reporting that the surface roughness morphology was well preserved after hydrothermal treatment when Mg was incorporated on titanium surfaces as well as that cell attachment and differentiation enhancement on surfaces treated with Mg [27].

In addition, Mg plays a part in various biological actions, including cell proliferation and differentiation as well as in the usual functionality of organs [28]. In the present study, Mg 1.4 wt% was achieved with 5 mM MgO after 3 h of hydrothermal synthesis. It could be dropped to 0.8 wt% and be closer to the levels seen in human bone by simply reducing the process to 2 h with the same settings (data not presented). 

It is essential to note that previous studies reveal that MgO ions at a concentration of roughly 2 mM can significantly stimulate the proliferation and differentiation of pre-osteoblasts and the upregulation of osteogenic genes in vitro and more new bone formation in vivo [10]. Nevertheless, MgO concentrations beyond 5 mM have destructive effects on human osteoblast differentiation and osseous metabolism, leading to bone mineralization defects (Table 1) [29,30].

There is no significant effect of MgO incorporation on the β-TCP structure on the changes in the lattice parameters detected by XRD, contrary to what was found in a study where Mg^2+^ was doped at the phase transformation temperature of β-TCP ceramics. Because of the substitution by Mg^2+^ on Ca^2+^, a higher peak structure was noticed at around 28° [15]. 

Generally, for the β-TCP stoichiometric dominant crystalline characteristics, intense sharp peaks were observed for both TCP particle phases [31]. In addition, our results indicate that the surface characteristics of β-TCP mediate osteoclastic activity via calcium ion saturation [32,33,34].

The FTIR results reinforced the XRD findings by presenting similar high-intensity peaks at the 1043 and 1120 cm^−1^ absorption bands in the β-TCP/Mg and untreated β-TCP particles [35]. Furthermore, both materials were probed to be apatites with similar crystalline phases and indicated that the hydrothermal synthesis of β-TCP/Mg had no new phase(s) formed during the process.

In vitro cytotoxicity and proliferation of MG-63 at 1, 7, and 21 days of culture in β-TCP/Mg and untreated β-TCP media behaved according to ISO10993-5. β-TCP/Mg improved MG-63 cell proliferation and displayed excellent biocompatibility with a statistically significant difference compared to cells maintained in nontreated beta-tricalcium phosphate and control cells in the present study. A significantly similar enhancement of human bone marrow mesenchymal stem cells (hBMSCs) and human umbilical vein endothelial cells adhesion and spreading was also found when Mg was doped to β-TCP at 1 and 2 wt%, similar to the 1.4 wt% we had in our EDS results [10]. In addition, both β-TCPs showed Grade 0 cytotoxicity, which suggests good cytocompatibility and acceptable biosafety for cellular applications [19]. However, at only 21 days, the cells that had been cultured with untreated β-TCP had proliferated significantly more than control cells (*p* < 0.0001). MG-63 osteoblast differentiation was also determined via qPCR, targeting established markers. During the initial phases of the osteogenic differentiation in the mesenchymal stem cells (MSC) in vitro, ALP and BSP gene expression were higher in the cells maintained in the beta-TCP and beta-TCP/Mg conditions than the control cells. Researchers have also demonstrated that the osteogenesis-related gene expression levels of ALP and BSP were also higher in hBMSCs when they cultured at similar concentrations of Mg-doped to β-TCP to the ones used in the present study, [10]. Note that OC expression was greater in the control group than it was in the other two groups; however, the difference did not meet the level of significance. This gene is expressed by mature osteoblasts [36]. Osteoclast differentiation suppression can be attributed in part to OPG expression, which did not differ significantly in expression between the three groups. Overall, gene expression indicated that the osteogenic differentiation of MG-63 cells started as early as 7 days. Mg ions are valuable for osteogenic differentiation [27]. Nonetheless, high concentrations of Mg ions reduce osteoblast differentiation and inhibit the expression of specific osteogenic markers [18], as seen with the gene expressions in this study.

In vivo, the β-TCP/Mg particle grafts started creating new bone while degrading critical calvaria size defect healing in New Zealand white rabbits from weeks 2 to 8, as seen in the micro-CT results. During week two, the bone formation defects treated with the beta-TCP control and beta-TCP/Mg were significantly superior compared to the control defects (*p* < 0.05). Then, during week 6, statistically significant differences were noted between both beta-TCP/Mg and beta-TCP (*p* < 0.01) with the β-TCP/Mg and control group (*p* < 0.05). Furthermore, at eight weeks, β-TCP/Mg induced 32.65% ± 1.24% bone regeneration. In addition, new bone formation was significantly more extensive in the β-TCP/Mg group than it was in the β-TCP control or control group (*p* < 0.05).

The histomorphometric results were more accentuated than the micro-CT results were but had the same tendencies. Analyses of the second week mainly revealed granulation tissue with minimal immature bone with inflammatory cells, osteoclasts, and a few osteoblasts. The small amount of regenerated immature bone in the defects filled with both sorts of β-TCP was in direct connection with the particles. The β-TCP particles induced immature bone regeneration through osteoconduction by serving as scaffolds between the borders of the defects and the particles in the middle of the defects. The control group regenerated new bone; however, it did not differ significantly from the beta-TCP control group and the beta-TCP/Mg group (*p* < 0.05). The β-TCP control generated more new bone than the control. Additionally, beta-tricalcium phosphate/Mg regenerated more new bone than that generated by the control (*p* < 0.001) and β-TCP control (*p* < 0.05) defects. 

From week 8 onwards, the r graft particles started reabsorbing while regenerating new bone, and s because of the particle scaffold characteristics, new bone began to mature. The β-TCP/Mg had better bone regeneration over the β-TCP control (*p* < 0.05), and control (*p* < 0.001). Additionally, the control defects did not close, and they maintained the space that they took up. In contrast, the β-TCP/Mg and untreated β-TCP particles managed to yield greater defect closure and space maintenance at eight weeks. These new bone and resorption results are in partial agreement with the results of other studies involving rabbit calvarial models [37]. Some reported that woven bone formation could be seen in the early weeks and that at around eight weeks more mature bone appeared at the borders of the defects due to the higher bone turnover rate in rabbi calvarial models, creating a mixture of woven and lamellar bones in the central region [38,39]. However, other studies have reported no significant signs of β-TCP graft degradation after eight weeks of rabbit calvarial defect healing [40]. This variation in results could be associated with the different particles sizes used in the studies. In our study, the same 500 µm–1000 µm β-TCP particle size was used for both the control and test groups, indicating the advantage of adding Mg to the particle surface. On average, the new bone area was 4% larger for critical defects treated with β-TCP/Mg (histologic new bone formation: 21.68% ± 4.51%; micro-CT new bone formation: 32.65 ± 1.24) than those treated with the β-TCP control (histologic new bone formation: 16.9% ± 3.60%; micro-CT new bone formation: 28.98 ± 2.65) (*p* < 0.05). The β-TCP that was used in this study has been used in a range of bone surgeries in the dentistry and medical orthopedic fields [8]. The time required for its resorption varies between 6 months and 12 months, and it is then replaced by newly formed osseous tissue [41]. After a healing period of 6 to 8 months in maxillary sinus procedures, this same β-TCP seemed to indicate that it is a biocompatible and osteoconductive material that can be used successfully as a bone substitute both histologically and histomorphometrically [42]. It produced 28.2% new bone with 32.9% residual graft particles. These percentages could be improved by adding Mg to the material through hydrothermal synthesis after the fabrication process, which would not modify the particles.

In summary, the hydrothermal method can be used as an effective and facile method to improve the evolution of biomaterials for bone tissue engineering treatment. The present results indicate that the hydrothermal addition of 1.4 wt% MgO to the particle surface of β-TCP particle significantly increased cell proliferation and osteoblastic differentiation in vitro and resulted in more new bone regeneration from histologic and micro-CT evaluation in vivo compared to the β-TCP control particles; altogether, Mg was advantageous to commercial β-TCP bone regeneration. However, further clinical studies will be required to assess the formation of new blood vessels, proliferation through porous surfaces, cellular attachment, and bone regeneration.

## 4. Materials and Methods

### 4.1. In Vitro

#### 4.1.1. Preparation of β-TCP

Β-TCP (Cerasorb M^®^, CURASAN Co Ltd., Frankfurt, Germany) with an interconnecting open multiporosity with micro, meso, and macro pores (5 µm–500 µm) and a granulation size of 500 µm–1000 µm of approximately 65% ± 5% total porosity was used. The irregularly polygonal-shaped β-TCP granules facilitate canting and interlocking to prevent micromovements [43,44]. Samples of both β-TCP control and β-TCP/Mg that were one gram in size were prepared for each test.

#### 4.1.2. Hydrothermal Process

To hydrothermally add MgO to the β-TCP particle surface at 150 °C in a 40 mL of Mg solution containing 0.2 M NaOH + 5 mM MgO for 3 h, the β-TCP particles were treated.

#### 4.1.3. Characterization of Surface Morphology

The particle surface morphologies were observed at an accelerating voltage of 15 kV using a Field-Emission Scanning Electron Microscope (SEM; Horiba, EX-250, Kyoto, Japan). Before SEM observation, both β-TCP types of particles were blanketed with Pd–Au layers that were 25 nm thick and were placed in a sputtering apparatus and scaled on an aluminum stub using carbon tape (IB-2, Hitachi Ltd., Tokyo, Japan).

#### 4.1.4. Energy-Dispersive Spectrometry

The SEM machine was equipped using an energy-dispersive X-ray spectrometer (EDS; EX-250, HORIBA, Kyoto, Japan). The s quantitative analysis of the surface elements on the particles before and after the addition of Mg was conducted by measuring the ratios of the emission X-ray lines that were obtained from the elements Mg, Ca, and P.

#### 4.1.5. X-ray Photoelectron Spectroscopy

X-ray photoelectron spectroscopy (XPS) spectra were used to characterize the surface chemical composition of β-TCP/Mg and the control. Briefly, a focused monochromatic Al-Kα X-ray (1486.6 eV) was employed as an excitation origin. The elemental spectra of C1s, O1s, N1s, Ca2s, and P2p were monitored with 220 W of power and a 15 kV running voltage. The binding energy (285 eV) was registered in survey scan spectra at 0.1 eV steps, and the pass energy was arranged at 140 eV (PerkinElmer Phi 5500 ESCA system) [45,46].

#### 4.1.6. X-ray Diffraction Analysis

X-ray diffraction (XRD, Panalytical X’Pert^3^ PRO, Malvern Panalytical Co. Ltd., Almelo, The Netherlands) measurements were used to estimate the chemical compositions and the crystallographic configuration of both the control and test β-TCP particles. The powder was analyzed with Ni-filtered CuKα radiation (40 kV, 30 mA). The powders were observed from 3–100° (2θ) and at a scan speed of 2°/min. The peaks that were obtained were compared to those of the standard references. 

#### 4.1.7. Fourier-Transform Infrared Spectroscopy

A Nicolet iS50 Fourier Transform Infrared (FTIR) spectrometer (Thermo Fisher Scientific, Waltham, MA, USA) was used in conjunction with an attenuated total reflection system to identify the components of the β-TCP/Mg specimens and the β-TCP controls. Note that FTIR spectra were obtained under the following parameters: wavelength range (4000 cm^−1^–400 cm^−1^), resolution (4 cm^−1^), temperature (25 °C), and humidity (65% ± 5%). A background spectrum was used to normalize the spectra. In addition, a dry system was employed to eradicate atmospheric moistness [47].

#### 4.1.8. Cell Culture

MG-63 cells were obtained from the Cell Cultures of the Bioresource Collection and Research Center Collection (Hsinchu, Taiwan). Following a previous protocol [48], MG-63 cells were maintained in HyClone Dulbecco’s modified Eagle’s medium (DMEM; HyClone Laboratories Inc., Logan, UT, USA) with penicillin–streptomycin (1%), fetal bovine serum (10%), and l-glutamine (4 mmol/L) under humidified conditions with 5% CO_2_ at 37 °C. The experimental material was evaluated using both indirect and direct contact. For the indirect contact, the concentration of the test cells was 1 × 10^4^ cells/mL in a 24-well cell culture plate (Nunclon; Nunc, Roskilde, Denmark). The β-TCP/Mg specimens and β-TCP controls were cultured in DMEM (1 g/10 mL) for one day, after which the medium was substituted with beta-TCP/DMEM or beta-TCP/Mg DMEM. The same DMEM was used for the control. The test group was separated into two parts: the effect of the liquid extract on the cells (WST-1) and the immediate linkage with the material on the cells (immunofluorescence).

#### 4.1.9. Cell Viability (WST-1)

MG63 cells were seeded for indirect contact viability testing. After one day of culture, the medium was aspirated, and the previously described fresh medium that was used in the cell culture was added on days 1, 7, and 21 after being incubated. Proliferation and viability were assessed using WST-1 reagent in accordance with the manufacturer’s instructions (Roche Applied Science, Mannheim, Germany). Briefly, two hundred microliters of culture medium were repositioned to a 96-well microtiter plate; an amount of 10 μL of WST-1 was counted to each well and incubated for one hour, vibrated gently for 1 min, and the absorbance value, which is proportionate to the cell viability of the cells, was estimated using an ELISA reader with a wavelength of 450 nm (Thermo Fisher Scientific Inc., San Jose, CA, USA) [49,50].

#### 4.1.10. Immunofluorescence

After cell culture, the MG-63 cells were experimented with using direct contact immunofluorescence microscopy to observe the cell distribution on the scaffolds. The technique has been previously defined, and each step was performed according to the manufacturer’s instructions [48]. Briefly, cells cultured on the control and test β-TCP particles and were washed, fixed, and permeabilized. Later, nuclear counterstaining was conducted using DAPI (0.1 μg/mL; 4′,6-diamidine-2′-phenylindole dihydrochloride; D9542 Sigma-Aldrich Chemie GmbH, Deisenhofen, Germany) for 10 min; subsequently, after staining, 200 μL of phalloidin solution was counted for 15 min. Finally, labeled particles were investigated in 24-well plates operating an Fv-1000 laser scanning confocal microscope (Olympus Corporation, Japan) in conjunction with a high power 40× objective lens and analyzed by Leica LASX software [51].

#### 4.1.11. Alkaline Phosphatase Assay

In order to evaluate the osteoblast differentiation, the ALP assay kit (1-Step p-Nitrophenyl Phosphate Disodium Salt (PNPP); Fisher Scientific, Waltham, MA, USA) was used according to the manufacturer’s protocols. Cells were cultured on media for durations of 1, 7, or 21 days, whereupon they were washed twice using PBS and detached using Triton-100 (300 μL, 0.05%). Next, they were centrifuged at 13,000 rpm for 3 min to remove any insoluble material, and a 400-μL aliquot of assay buffer was added to each sample. Next, 80 μL of the solution was added to a 96-well plate. Finally, one hundred microliters of 1-Step PNPP were combined with each well and gently blended. Afterward, incubation was carried out at room temperature (25 °C) for 60 min followed by the addition of 0.4 M NaOH stop solution. The optical density at 405 nm was determined using the same ELISA reader as the one used in the WST-1 test [52].

#### 4.1.12. Real-Time Polymerase Chain Reaction

The real-time polymerase chain reaction (PCR) analysis was performed on alkaline phosphatase (ALP), osteocalcin (OC), bone sialoprotein (BSP), and osteoprotegerin (OPG) genes and was normalized using human glyceraldehyde-3-phosphate dehydrogenase. The real-time PCR assay process was conducted as defined in previous investigations [48,53,54]. We analyzed the differentiation effectiveness of the undifferentiated cells compared to the effectiveness of the control groups at days 1, 7, and 21. First, the total RNAs (Total RNA Mini Kit) were isolated according to the manufacturer’s protocols (NOVELGENE, Molecular Biotech Corporation, Taiwan). Next, the mRNA of cells medium (DMEM) + beta-TCP control or beta-TCP/Mg medium were changed into cDNA utilizing the cDNA Synthesis Kit (Thermo Fisher Scientific Company, Waltham, MA, USA). Then, qPCR was conducted on the cDNA samples by employing the Fast SYBR™ Green Master Mix (Thermo Fisher Scientific Baltics UAB, Vilnius, Lithuania). We used primer sets with a final concentration of 0.3 mM and a final 1.5 mL/tube volume. To obtain thermal profiles using 2 μL cDNA samples within a 20 μL reaction volume, we used the Roche Real-Time PCR System (LightCycler^®^ 96 instrument, Roche Molecular Systems, Inc., Pleasanton, CA, USA). We then performed PCR at 95 degrees Celsius for 10 min and then subsequently performed 40 cycles at 95 degrees Celsius for 15 s and at 60 degrees Celsius for 1 min. Quantification was performed utilizing the delta–delta calculation technique [48,55].

### 4.2. In Vivo Analysis

#### 4.2.1. Surgical Procedure

Fifteen adult male New Zealand white rabbits weighing 2.1 kg with an average age of 3 months old were used in this study. According to animal experiment (ARRIVE; Animal Research Reporting In Vivo Experiments) guidelines, the animals were held in separate cells in a temperature-controlled environment at the Animal Center. Water and food were freely accessible to the animals. First, Zoletil 50 anesthesia (50 mg/mL) was injected intramuscularly at 15 mg/kg/dose in the gluteal area. Then, a local anesthetic solution (1.8 mL, 2% lidocaine with epinephrine 1/100,000) was used in the periphery of the calvaria. A deep incision that was 2 cm in length was made in the midline of the skull. The periosteum was ablated, and three full-thickness 6.0-mm-diameter calvarial bone defects were created in the rabbits using a sterile bone trephine bur (3i Implant Innovation Inc., Palm Beach Gardens, FL, USA) under constant saline buffer irrigation [56]. Randomly, HA/β-TCP controls and HA/β-TCP/Mg were used to fill the defects, and control defects were left to heal spontaneously [57,58,59,60].

#### 4.2.2. Sample Preparation

Two, six, and eight weeks after surgery, five animals were sacrificed randomly through carbon dioxide inhalation for 10 min. Then, calvarial bones were dissected and fixed instantly in 10% *v/v* buffered formalin for analysis. 

#### 4.2.3. Micro-Computed Tomography of New Bone and Tissue Area

The bone regeneration levels observed in the samples were assessed using micro-computed tomography (µCT, SkyScan 1176, Bruker Micro-CT, Kontich, Belgium). First, the recently developed bone in the defect zone was calculated using an imaging software program (Data Viewer, CT-Analyser, Bruker Micro-CT, Kontich, Belgium). After calibration, the image files were opened in the imaging software program, and the entire defective coronal region was selected. Afterward, all of the pixels within this region with a grayscale weight over a predefined threshold between units 20 and 80 corresponding to the mineralized bone were measured and quantified as the “new bone formation site.” Additionally, the total defect volume was estimated from the µCT. Finally, the percentage of recently formed bone in each defect region was assessed using the following formula: The portion of the newly formed bone (%) equals newly formed bone volume (μm^3^) divided by total defect volume (μm^3^) [61].

#### 4.2.4. Histomorphometric Analysis

Specimens were fixed in 10% *v*/*v* buffered formalin to observe the mineralization process fixed for 14 days, the solution was renewed every 24 h, and the samples were decalcified by immersion. Afterward, the samples were cleared with toluene and dehydrated in a graded series of ethanol solutions (80%–100%). Each sample was implanted in paraffin using the standard method; specimens were sectioned into 5 µm (Leica Microsystems SP 1600, Wetzlar, Germany) and shaded with hematoxylin and eosin (H&E). The histomorphometric evaluation of new bone area/tissue area percentage comprised an examination of a midsection slide through the calvaria defects at 40× magnification (ImageJ program, National Institutes of Health, NIH; Bethesda, MD, USA).

#### 4.2.5. Statistical Analysis

All of the experimental groups were performed in triplicate, and the study results were described as means ± standard deviation (SD). Student’s t-test was utilized to determine the differences between the groups (Microsoft Software, Redmond, WA, USA). *p* values of <0.05 were considered statistically significant.

## 5. Conclusions

Our study suggests that the addition of 5 mM MgO on β-TCP particles after 3 h of hydrothermal synthesis significantly increased MG-63 cell proliferation and osteoblastic differentiation in vitro, simultaneously regenerating more new bone through osteoconduction in vivo without altering the surface roughness.

## Figures and Tables

**Table 1:** Elemental results by EDS (Energy-Dispersive X-ray Spectrometry).

Chemical Element	β-TCP/Mg (Test)	β-TCP (Control)
	Weight (%)	Weight (%)
Mg	1.4	0.0
Ca	35.9	39.2
P	19.7	20.8
O	43.0	40.1
Ca	0.0	0.0

**Table 2:** XPS analyses (%).

Group	O1s	Na1s	Mg1s	Si2p	P2p	S2p	Ca2p	Mg/Ca	P/Ca	P/Ca + Mg
β-TCP/Mg	48.43	3.67	2.08	0.74	8.79	0.39	12.36	16.80%	71.10%	60.90%
β-TCP	49.74				11.8	0.83	16.36	0.00%	72.10%	72.10%

**Table 3:** Bone formation by Microcomputed Tomography scan (μCT; %).

	Week Two	Week Six	Week Eight
β-TCP/Mg	22.73 ± 5.39	28.36 ± 1.70	32.65 ± 1.24
β-TCP	21.74 ± 4.24	24.73 ± 4.41	28.98 ± 2.65
Control	14.54 ± 5.23	15.58 ± 5.45	24.1 ± 4.05

**Table 4:** Histology of newly formed bone (%).

	Week Two	Week Four	Week Eight
β-TCP/Mg	11.48 ± 4.88	24.42 ± 4.53	31.62 ± 3.03
β-TCP	10.42 ± 4.89	15.07 ± 7.91	24.94 ± 3.00
Control	10.14 ± 2.11	12.73 ± 3.26	19.73 ± 3.37

## Data Availability

Data are contained within the article.
